# A multisite analysis of the concordance between visual image interpretation and quantitative analysis of [^18^F]flutemetamol amyloid PET images

**DOI:** 10.1007/s00259-021-05311-5

**Published:** 2021-04-12

**Authors:** Marco Bucci, Irina Savitcheva, Gill Farrar, Gemma Salvadó, Lyduine Collij, Vincent Doré, Juan Domingo Gispert, Roger Gunn, Bernard Hanseeuw, Oskar Hansson, Mahnaz Shekari, Renaud Lhommel, José Luis Molinuevo, Christopher Rowe, Cyrille Sur, Alex Whittington, Christopher Buckley, Agneta Nordberg

**Affiliations:** 1 Division of Clinical Geriatrics, Center for Alzheimer Research, Department of Neurobiology, Care Sciences and Society, Karolinska Institutet, Stockholm, Sweden; 2 Medical Radiation Physics and Nuclear Medicine, Section for Nuclear Medicine, Karolinska University Hospital, Stockholm, Sweden; 3 Pharmaceutical Diagnostics, GE Healthcare, Amersham, UK; 4 Barcelonaβeta Brain Research Center (BBRC), Pasqual Maragall Foundation, Barcelona, Spain; 5 IMIM (Hospital del Mar Medical Research Institute), Barcelona, Spain; 6 Department of Radiology and Nuclear Medicine, Amsterdam UMC, Vrije Universiteit Amsterdam, De Boelelaan, 1117 Amsterdam, Netherlands; 7 Austin Health, University of Melbourne, Melbourne, Australia; 8 Health and Biosecurity, CSIRO, Parkville, Australia; 9 Universitat Pompeu Fabra, Barcelona, Spain; 10 Centro de Investigación Biomédica en Red Bioingenieriá, Biomateriales y Nanomedicina, (CIBER-BBN), Barcelona, Spain; 11 Invicro, London, UK; 12 Division of Brain Sciences, Department of Medicine, Imperial College, London, UK; 13 Neurology and Nuclear Medicine Departments, Saint-Luc University Hospital, Av. Hippocrate, 10, 1200 Brussels, Belgium; 14 Gordon Center for Medical Imaging, Department of Radiology, Massachusetts General Hospital, Harvard Medical School, Boston, MA USA; 15 Clinical Memory Research Unit, Department of Clinical Sciences Malmo, Lund University, Lund, Sweden; 16 Centro de Investigación Biomédica en Red de Fragilidad y Envejecimiento Saludable (CIBERFES), Madrid, Spain; 17 Department of Medicine, The University of Melbourne, Melbourne, Australia; 18 Merck & Co., Inc., Kenilworth, NJ USA; 19 Department of Aging, Karolinska University Hospital, Stockholm, Sweden

**Keywords:** Amyloid PET, [^18^F]flutemetamol, Image interpretation, Visual inspection, Quantification, Alzheimer’s disease

## Abstract

**Background:**

[^18^F]flutemetamol PET scanning provides information on brain amyloid load and has been approved for routine clinical use based upon visual interpretation as either negative (equating to none or sparse amyloid plaques) or amyloid positive (equating to moderate or frequent plaques). Quantitation is however fundamental to the practice of nuclear medicine and hence can be used to supplement amyloid reading methodology especially in unclear cases.

**Methods:**

A total of 2770 [^18^F]flutemetamol images were collected from 3 clinical studies and 6 research cohorts with available visual reading of [^18^F]flutemetamol and quantitative analysis of images. These were assessed further to examine both the discordance and concordance between visual and quantitative imaging primarily using thresholds robustly established using pathology as the standard of truth. Scans covered a wide range of cases (i.e. from cognitively unimpaired subjects to patients attending the memory clinics). Methods of quantifying amyloid ranged from using CE/510K cleared marked software (e.g. CortexID, Brass), to other research-based methods (e.g. PMOD, CapAIBL). Additionally, the clinical follow-up of two types of discordance between visual and quantitation (V+Q- and V-Q+) was examined with competing risk regression analysis to assess possible differences in prediction for progression to Alzheimer’s disease (AD) and other diagnoses (OD).

**Results:**

Weighted mean concordance between visual and quantitation using the autopsy-derived threshold was 94% using pons as the reference region. Concordance from a sensitivity analysis which assessed the maximum agreement for each cohort using a range of cut-off values was also estimated at approximately 96% (weighted mean). Agreement was generally higher in clinical cases compared to research cases. V-Q+ discordant cases were 11% more likely to progress to AD than V+Q- for the SUVr with pons as reference region.

**Conclusions:**

Quantitation of amyloid PET shows a high agreement vs binary visual reading and also allows for a continuous measure that, in conjunction with possible discordant analysis, could be used in the future to identify possible earlier pathological deposition as well as monitor disease progression and treatment effectiveness.

**Supplementary Information:**

The online version contains supplementary material available at 10.1007/s00259-021-05311-5.

## Introduction

[^18^F]Flutemetamol is a PET amyloid imaging agent approved for diagnostic assessment of amyloid deposits in the brain [1, 2] and for ruling out the presence of Alzheimer disease (AD) pathology in subjects with cognitive complaints [3]. The tracer has been robustly validated against accepted CERAD pathology measures as the standard of truth [4], with both sensitivity for measuring the PET signal equating to moderate and frequent neuritic amyloid plaque density and specificity for excluding none or sparse neuritic amyloid being over 90% [5, 6]. Methods for the visual interpretation of [^18^F]flutemetamol scans as either negative (none or sparse amyloid) or positive (moderate or frequent amyloid) were formulated using scans collected in a phase II study [7] and then developed into an electronic image read training program using a large cohort of images from a variety of sources (i.e. from healthy young and older volunteers, mild cognitive impairment (MCI), and AD dementia) as the test sample [8]. Training programs are available prior to physician use in routine clinical practice in order to optimize the accurate reading of the PET images [9].

Inspection of the [^18^F]flutemetamol image entails the assessment of 5 specific regions of the brain which are known to accumulate pathology, i.e. frontal, temporal, posterior cingulate/precuneus, temporo-parietal and striatum [8]. Any one of these regions can be read as positive unilaterally for the whole scan to be deemed positive and a negative scan should have no discernible PET signal in any of these regions. An analysis of cases from both a clinical follow-up study in amnestic MCI subjects and the pivotal end-of-life autopsy study showed in the majority of cases (over 90%) [^18^F]flutemetamol images were positive in 4–5 regions and cases with only 1–2 regions positive were limited [10].

[^18^F]Flutemetamol and other amyloid PET tracers were approved in 2013/2014 and therefore have been in routine use for over 5 years, with utility for changing diagnosis and increasing diagnostic confidence being demonstrated in many studies [11, 12]. Visual inspection was the method approved for image interpretation; however, in the intervening period, multiple image analysis software tools have become available which allow for the quantification of cortical amyloid load as both composite and regional measures. Although frequently used in the research setting, nuclear medicine physicians can also use these quantitative tools as an objective means to provide a numerical value which relates to pathological amyloid status. In CE marked software currently those units are either the SUVr or *Z* score. In order to be consistent with current practice, the manufacturer of [^18^F]flutemetamol received permission from the European regulators (EMA) to add the use of quantitation as an adjunct to visual reading which continues to be the primary method for image inspection [13].

Therefore, the post hoc analysis presented here examined data from a heterogenous range of sources to measure both the concordance and discordance between [^18^F]flutemetamol image reading and quantitative assessment. The agreement rate between these measures was included as a secondary objective in some of these cohorts and in others the data was newly generated. Additionally, other goals consisted of (1) a more detailed examination (including clinical follow-up where available) of discordant visual/quantitation cases where available, (2) a sensitivity analysis to examine agreement around the visual/quantitative threshold and (3) assessing the effect of reconstruction methods on the composite amyloid measure using equivocal [^18^F]flutemetamol images taken from the routine clinical setting.

The data generated in this multisite analysis could be valuable for radiologists in the routine clinical setting as updated regulatory instructions now include quantitative assessment in addition to visual inspection. This study aims to demonstrate to users who add quantitation to their [^18^F]flutemetamol image interpretation procedures the levels of concordance and discordance plus give further detail to those patterns of discordance. Experience is also drawn from the Karolinska University Hospital which has been performing these amyloid PET scans for over 5 years and routinely uses a combination of visual reading and quantitation when reporting scan results.

Other investigators have evaluated the value of quantitation in supporting the visual read of amyloid PET image interpretation [14–16] in smaller studies assessing up to 175 scans. The analysis in this paper extends these observations by including over 2700 images collected from a range of single and multicentre studies both from research and clinical populations as well as focusing on a detailed assessment of cases with discordance between the two methods of image interpretation.

## Methods

### [^18^F]Flutemetamol imaging

A total of 2770 image read results from 9 studies/research reports were collected where subjects or patients had been administered with approximately 185 MBq [^18^F]flutemetamol injection and PET imaged for approximately 20 min at 90 min post injection. All images were interpreted by nuclear medicine physicians or technologists trained with instructions provided by the manufacturer [9].

Visual read image analysis and study details (subjects imaged and quantitation software) were included in multisite analysis (Table 1).

1. **GE Healthcare Development Studies** (GE): 172 Vizamyl images (from Study GE-067-021, Clinical-Trials.gov identifier NCT01672827 comprising of 33 subjects with clinical probable Alzheimer’s disease (AD), 80 subjects with mild cognitive impairment, and 59 healthy volunteers) were visually read by 5 highly trained independent readers and a majority negative or positive image read recorded [17]. SUVr thresholds were calculated using autopsy pathology as the standard of truth. Quantification was performed using Cortex ID [17].
2. **Karolinska Institutet/Karolinska University Hospital (Stockholm, Sweden)** (KAROLINSKA): study, 207 patients with cognitive issues, who were undergoing routine clinical diagnosis, received a [^18^F]flutemetamol scan. Images were visually interpreted by two of three blinded readers (two highly trained and one moderately skilled) according to the instructions from GE Healthcare and consensus was reached by discussion in the case of disagreement. Twenty-one of the cases were considered borderline (i.e. not clear as either negative or positive) after visual read. Semiquantitative analysis was performed using BRASS software applying an SUVr threshold cut-off of 0.60 (reference region was pons) [18].
3. **Merck Study** (MCK): MK-8931 study, 928 amnestic mild cognitive impairment (aMCI) subjects underwent [^18^F]flutemetamol scanning in 17 countries as part of inclusion/exclusion criteria for a phase III Verubecestat trial [19, 26], in over 150 imaging centres using over 25 scanner models. One of two certified neuroradiologists interpreted the images as either normal or abnormal. All images were quantified with FreeSurfer open source software suite where the native space magnetic resonance imaging (MRI) regions of interest were applied on the co-registered PET data to calculate the SUVr values (https://surfer.nmr.mgh.harvard.edu/). The distribution of the SUVr pons data across the study population confirmed a similar cut-off of 0.62 to differentiate negative and positive scans as reported in Study 1 [19, 26].
4. **St Luc, Brussels** (Investigator Sponsored Study) (SLC): [^18^F]flutemetamol scans were acquired in 94 aMCI cases (routine sequential cases), 35 SCD and 31 healthy control subjects (*n* = 160) and images read visually by a single highly trained reader. Seven of the subjects were considered borderline after visual inspection. Scans were quantified using PMOD research (https://www.pmod.com/web/?page_id=53) software using a cortical composite and cerebellar grey matter as the reference region [20].
5. **Amsterdam University Medical Centre** (Investigator Sponsored Study) (AUMC): 145 clinical cases with available MRI and [^18^F]flutemetamol scans were read visually by 3 trained readers (one with high, one with moderate, and one with low level of training), and a majority vote decided the result. Quantitation was measured by SUVr with grey matter cerebellum as the reference region using whole cortex without occipital lobe, primary motor and sensory cortex. The cut-off for positivity was calculated for receiver operating curve (ROC) based on the cases that were read concordantly negative (*n* = 42) and concordantly positive (*n* = 89). SUVr measurements derived using the open source software based upon PVElab. (https://nru.dk/index.php/component/jdownloads/ category/37-pvelab) [21].
6. **Barcelona ALFA+ cohort** (Investigator Sponsored Study) (ALFA+): 361 subjects (all cognitively unimpaired) were scanned with [^18^F]flutemetamol and images visually read by 1 of 2 trained readers (one moderately and one experienced reader) using the Syngo.via viewer contained within the console of the Siemens PET scanner. Two of the subjects were considered borderline after visual inspection. SPM12 neuroimaging software (https://www.fil.ion.ucl.ac.uk/spm/software/spm12/) was used as the quantitative tool for this cohort [22, 27].
7. **Swedish Biofinder Lund** (Investigator Sponsored Study) (BIOFINDER): 401 [^18^F]flutemetamol images were collected from subjects with subjective cognitive decline (SCD) and mild cognitive impairment (MCI). Images were read independently by 3 trained readers with experience in neurology PET imaging and were blinded to clinical status, diagnosis and other biomarker information. Majority vote was defined as two of three readers in agreement. Scans were quantified using PMOD research (https://www.pmod.com/web/?page_id=53) software using a cortical composite region and cerebellar grey matter as the reference region [23].
8. **Invicro Read Study** (INVICRO). A pool of 120 random scans from BIOFINDER (above #7) were assessed independently by 3 blinded readers trained using the majority read as the comparative measure. Read results were compared to amyloid load estimates (Ab_Load_) from the Amyloid^IQ^ processing pipeline developed by Whittington and Gunn [24], which is an analytical tool for the quantification of amyloid levels that enables classification of subjects (Ab-/Ab+). Analyses on the PET data was performed in the absence of access to associated structural MRI data.
9. **AIBL study Melbourne** (Investigator Sponsored Study) (AIBL) [28]. A total of 276 subjects from the cohort of over 1100 (*n* = 189 controls [some with subject cognitive complaints], *n* = 65 mild cognitive impairment, *n* = 18 probable Alzheimer’s disease and *n* = 4 other Dementia) were scanned with [^18^F]flutemetamol and images read visually and classified as negative or positive by a highly experienced reader. Seven of the subjects were considered borderline (with lowest level of confidence in visual reading). Using software developed by CSIRO (Commonwealth Scientific and Industrial Research Organisation) in combination with AIBL (CapAIBL [29]) a composite SUVr was measured using pons as the reference region and a threshold consistent with that reported by Thurfjell et al. [17] was used for classification.

**Table 1:** Summary of studies and clinical populations used for the multisite analysis

Study_Main_Institute	Study_abbr.	Type	Population	Country	Study_Reference	Ref_regions	Region_delineation	Target_regions	Software	Total_cases
GE Healthcare/Imanet (Uppsala, Sweden)	GE	Clinical research	HC: 59, MCI: 80, (p)AD: 33	Denmark, Belgium, USA, others (WW)	Thurfjell L, et al. JNM 2014 [17]	Pons, CGM, WC	PET-only adaptive template	FL,TL_lat,Cing,PL*	Cortex ID	172
Karolinska Institutet (Huddinge, Sweden)	KAROLINSKA	Clinical routine	SCD: 5, MCI: 131, AD: 41, non-AD: 10, DemNOS: 20	Sweden (Europe)	Leuzy A, et al. EJNMMI 2019 [18]	Pons	PET-only automated ROI-based	FL,TL_lat,Cing,PL	BRASS	207
Merck and Co Inc. (c/o Bioclinica) (USA)	MCK	Clinical research	(a)MCI: 928	WW (17 Countries)	Sur C, et al. Brain 2020 [19]	Pons, WC	PET-MR automated ROI-based	FL, TL, PL, Cing, Precuneus	Freesurfer	928
Saint-Luc University Hospital (Brussels, Belgium)	SLC	Clinical routine	HC: 31, SCD: 35, MCI: 94	Belgium (Europe)	Hanseeuw B, et al. EJNMMI 2020 [20]	CGM, WC	PET-MR automated template	Neocortex	PMOD	160
Amsterdam UMC (Amsterdam, The Netherlands)	AUMC	Clinical routine	Early-onset dementia (<70 yrs): 145	Netherlands (Europe)	Zwan MD, et al. Alzheimer’s Research & Therapy 2017 [21]	WC	PET-MR automated ROI-based	FL, TL, PL	PVElab	145
Barcelona Brain Research Centre (BBRC) (ALFA+ Cohort) (Barcelona, Spain)	ALFA+	Clinical research	HC(ADO): 361	Spain (Europe)	Salvadó G, et al. Alzheimers Res Ther. 2019 [22]	Pons, WC	PET-only automated ROI-based	AAL Composite: FL, TL, PL, Cing, Precuneus, Angu-lar, Supram. CTX: Centiloid Global Cortical Average	SPM12	361
Lund University (BIOFINDER Cohort) (Malmö, Sweden)	BIOFINDER	Clinical research	HC: 134, SCD: 118, MCI: 149	Sweden (Europe)	Hansson O, et al. A&D 2018 [23]	Pons	PET-MR automated ROI-based	FL, TL_lat_post, Cing./Precuneus, PL	PMOD and NeuroMark	401
Invicro (Imaging Clinical Research) (London, UK)	INVICRO	Clinical research	Random sample from BIOFINDER: 120	Sweden (Europe)	Whittington A, et al. J Nucl Med 2019 (method) [24]	NA	ABLoad	NA	AmyloidIQ	120
AIBL (Australia)	AIBL	Clinical research	HC: 184, MCI: 60, AD: 18, non-AD: 3, unknown: 11	Australia	van der Kall LM, et al. Neurology 2020 [25]	Pons, WC	PET-only adaptive Atlas	FL, TL_lat, Cing, OL_lat	CapAIBL	276
									Total	2770

*Abbreviations*: *HC*, healthy control; *MCI*, mild cognitive impairement; *(p)AD*, probable AD; *SCD*, subjective cognitive decline; *AD*, Alzheimer’s disease; *non-AD*, non-AD dementia; *DemNOS*, dementia not otherwise specified; *ADO*, offspring of AD parent(s); *WC*, whole cerebellum; *CGM*, cerebellar grey matter; *WW*, worldwide; *NA*, not applicable; *Data Driven*, not derived from anatomical atlas; *FL*, frontal; *TL*, temporal; *OL*, occipital; *PL*, parietal; *lat*, lateral; *post*, posterior; *Cing*, cingulate; *Supram*, supramarginal; *Ctx*, cortex; *(minimizing spill-over from white matter, region named ‘Narrow’ in [17])

### Quantitative image analysis

Quantitative analysis was performed via a variety of methods. In two cases the software tool had a CE marked/510K approval (Cortex ID [17] or Hermes Medical Solutions Brass [30]) and in the other 7 cases the software tool was one frequently used in the research setting (Freesurfer [31], PMOD [32], PVElab [33], SPM12 [34], Amyloid^IQ^ [35] and CapAIBL [29]. Most importantly, for each software tool, the method for determining the threshold between a positive and negative result was recorded too for comparative purposes. In general, a composite cortical standard uptake value ratio (SUVr) was used as the measure to compare to the dichotomous visual read. Further details on the reference regions used are shown in Table 1. Most of the differing analysis in this study used pons as the reference region for the SUVr measure as the majority of images used this reference region (although where highlighted some of the cohorts used either whole cerebellum or cerebellar grey).

The fixed cut-off for positivity used for pons reference region (0.62) was primarily driven by the large multicentre Merck study (*n* > 900 clinical cases) generating this pons threshold/cut-off from their visual inspection data. Additionally the figure is consistent with the 0.61 validated by comparing visual and quantitative [^18^F]flutemetamol read data to pathological levels of neuritic amyloid in the pivotal phase III autopsy study [17]. An optimal cut-off has also been calculated for each study using pons as reference region as described in the following section.

### Sensitivity analysis

Sensitivity analysis was performed on the combined datasets where SUVr with pons as reference region and respective visual reads were available. The quantitation cut-off was incrementally shifted and percentual agreement between visual and quantitative methods computed. In order to include the peak agreement of all studies included, the final range of cut-off was from 0.55 to 0.73. The sensitivity analysis has been performed with the datasets with visual read borderline cases excluded as this was a negligible fraction of the overall dataset (<1% of total number of subjects, (*n* = 16, 7 and 2 for KAROLINSKA, AIBL and ALFA+ respectively)). Pons was used as the primary reference for this analysis as the region is consistently used in CE marked software and the region was extensively validated across CERAD amyloid pathology in autopsy studies as the standard of truth [16].

### Statistical analysis

#### Concordance and discordance analysis

The concordance of the visual read and quantitative results are presented in overall percentage terms with mean %, weighted mean %, SD, median and range for the stated reference regions. For the Pons reference region values for a fixed cut-off and an optimized cut-off based on sensitivity analysis (see above for methods) are presented.

For all 9 studies included, the total number of subjects discordant (i.e. V+Q- or V-Q+) using primarily the SUVr measure was determined; borderlines (<1% of the total number of cases) were excluded. The information was available for all studies, apart from the MCK study (#3), where discordances were derived from reference [26], by analysing the Gaussian mixture modelling of the SUVr of the V+ and V- groups.

Where possible the discordancy analysis also included a clinical follow-up to assess the longer-term prognosis of these cases (only see below for methods).

### Follow-up analysis on discordant cases

Follow-up data on any clinical progression compared to the baseline visit with [^18^F]flutemetamol PET was collected from 5/8 cohorts (KAROLINSKA, SLC, AUMC, BIOFINDER, AIBL). The diagnoses were classified as either to AD or other diagnosis (including SCD, MCI, non-ADD, vascular diseases, Parkinsonian diseases (Parkinson disease and progressive supranuclear palsy), normal pressure hydrocephalus).

The follow-up data was available for the discordant cases of KAROLINSKA, BIOFINDER and AIBL studies for the pons reference region and for SLC, AUMC and AIBL for the whole cerebellum (WC) reference region. The follow-up time was restricted to 4 years (rounded) for the pons and 3 years (rounded) for the WC according to the availability of data.

The comparisons between the two different discordant profiles (either V+Q- or V-Q+), borderline profiles (either BL/Q+ or BL/Q-) and the progression to a worse clinical diagnosis (AD + other diagnosis) or AD vs other diagnosis (OD) have been tabulated. Pons reference region results are presented for both fixed and optimized cut-off. Competing risk regressions (CRR) to test whether V+Q- and V-Q+ profile differ between AD or OD discounting for the probability of the other event to occur was performed for the pons reference region with fixed cut-off for which there was a significant number of discordant cases for the analysis. For the CRR analysis, the full follow-up dataset available (up to 7 years) and censoring information was taken in consideration [36], similarly to what is usually performed in a survival analysis.

For all statistical analysis, R version 4.0.2 was used.

### PET image reconstruction parameter analysis

Supp Table 1 describes in detail the scanner models and reconstruction types and parameters of the studies included in this work. For the KAROLINSKA cohort, we also compared three sets of frequently used PET reconstruction parameters for both the discordant cases and (visually and SUVr cut-off) borderline cases (*n* = 21 were available for the 3 sets of parameters) across the range 0.57–0.62 with pons as the reference region and measured the composite amyloid SUVr using Hermes BRASS software. The reconstruction parameters utilized were (a) 128 × 128, Gaussian filter 3 mm (Ref-128, reference, also chosen for the main analyses), (b) 256 × 256, Gaussian filter 3 mm (alternative 1, Alt-256) and (c) 400 × 400, Gaussian filter 2 mm (alternative 2, Alt-400).

To evaluate the impact of two different pipelines with the same reference region (Pons), we have performed the sensitivity analysis on the ALFA+ dataset with two pipelines: one quantifying SUVr values in the standard Montreal Neurological Institute (MNI) space using the Centiloid Global Cortical Average ROI (http://www.gaain.org/centiloid-project) and another using as target region a composite of regions from the AAL Atlas (https://www.gin.cnrs.fr/en/tools/aal/) that have been brought to the subject’s space and masked with a grey matter parcellation.

## Results

### 1) Cohort description

Three of the cohorts (KAROLINSKA, SLC, AUMC) comprised of subjects with cognitive complaints collected primarily from clinical routine whilst the remaining 6 (GE, MCK, ALFA+, BIOFINDER, INVICRO, AIBL) comprised of research studies covering a wider range of subjects from cognitively unimpaired (CU), subjective cognitive decliners (SCD), mild cognitive impairment (MCI) to dementia due to AD. The pooled cohort consisted of 769 (28%) CU subjects, 158 (6%) SCD, 1442 (52%) MCI, 237 (9%) dementia due to AD, 33 (1%) dementia due to non-AD and 131 (5%) of unknown diagnosis (missing data) (*n* = 2770 total).

For 6 out of 9 studies (Table 1), both pons and whole cerebellum reference regions (RR) were available, cerebellar grey matter RR was available for 2 out of 9 studies (GEHC and Amsterdam UMC), and for one study amyloid load was used (Invicro) (Table 1).

### 2) Assessment of agreement between visual inspection and quantitation

A summary of the agreement rates for the various studies with the different RRs used is shown in Table 2. Agreement rates for all reference region/methods ranged from approximately 88 to 100% depending upon population studied and reference region assessed. Generally, most results lie in the 93–99% range, indicating the comparability/generalizability between visual inspection and quantitation irrespective of the software/camera type/image reconstruction methodology employed. Inclusion of borderline cases and computation of either weighted or arithmetic mean did not significantly influence the agreement rate. A higher agreement rate was observed for the whole cerebellum region with a mean of 96% (the cut-offs available were slightly different). The agreement for the pons region was in average 94% when the fixed SUVr cut-off was used (Table 2) or 96.5% and 95.5% (mean and weighted mean, respectively) when the optimized SUVr cut-offs were used (Table 2). The cerebellar grey matter resulted in a slightly higher percent agreement (98.8%). The Invicro amyloid load method showed a 92.5% agreement.

**Table 2:** Summary of the percentage agreement between visual interpretation and quantitation across study groups using fixed and optimized cut-offs

Study Abbr.	RR Pons (agreement, %)	RR Pons (cut-off)	RR WC (agreement, %)	RR WC (cut-off)		RR CGM (agreement, %)	RR CGM (cut-off)	ABload (agreement, %)		ABload (cut-off)
GE	99.4% 97.7%	0.59 (Optim) 0.62 (Fixed)	98.8%	1.25		98.3%	1.57			
KAROLINSKA	100% 99.0%	0.60 (Optim) 0.62 (Fixed)								
MCK	94.4%	0.62 (Optim/Fixed)	94.4%	1.2						
SLC		99.3%		1.35	99.3%		1.56			
AUMC			96.6%	1.22						
ALFA+	93.3% 88.4%	0.57 (Optim) 0.62 (Fixed)	94.2%	1.24						
BIOFINDER	96.5% 91.8%	0.7 (Optim) 0.62 (Fixed)								
INVICRO								92.5%	33	
AIBL	95.2% 94.4%	0.65 (Optim) 0.62 (Fixed)	95.2%		1.2					
Mean	96.5% 94.4%*	Optim Fixed	96.4%			98.8%				
Mean (weighted)	95.5% 93.8%*	Optim Fixed	95.4%			98.8%				
SD	2.7% 3.6%	Optim Fixed	2.2%			0.8%				
SD (weighted)	2.1% 2.7%	Optim Fixed	1.7%			0.5%				
Median	95.8% 94.4%	Optim Fixed	95.9%			98.8%				

Borderline cases were excluded from the calculations. Where available study agreement using SUVr by different reference regions is presented. For 5/6 studies reporting pons reference region, raw data was available to perform sensitivity analysis and find optimized cut-offs. The fixed pons threshold with a cut-off of 0.62 was used as validated via autopsy correlation with PET imaging [17] and modelling of the large Merck dataset [26], and this was the dataset not available for further sensitivity analysis. *T*-test comparing fixed and optimized cut-off was performed on both unweighted and weighted data for pons reference region. *p < 0.05. *RR*, reference region; *WC*, whole cerebellum; *CGM*, cerebellar grey matter

### 3) Sensitivity analysis around the visual cut-off

A sensitivity analysis, comparing visual read results to a range of cut-offs around the autopsy validated threshold [17] (pons threshold approximately 0.58–0.62 as used in CE marked software such as Cortex ID or Hermes Brass), was performed. Five datasets were available (GE, KAROLINSKA, AIBL, Biofinder and ALFA+).

**Fig. 1:**
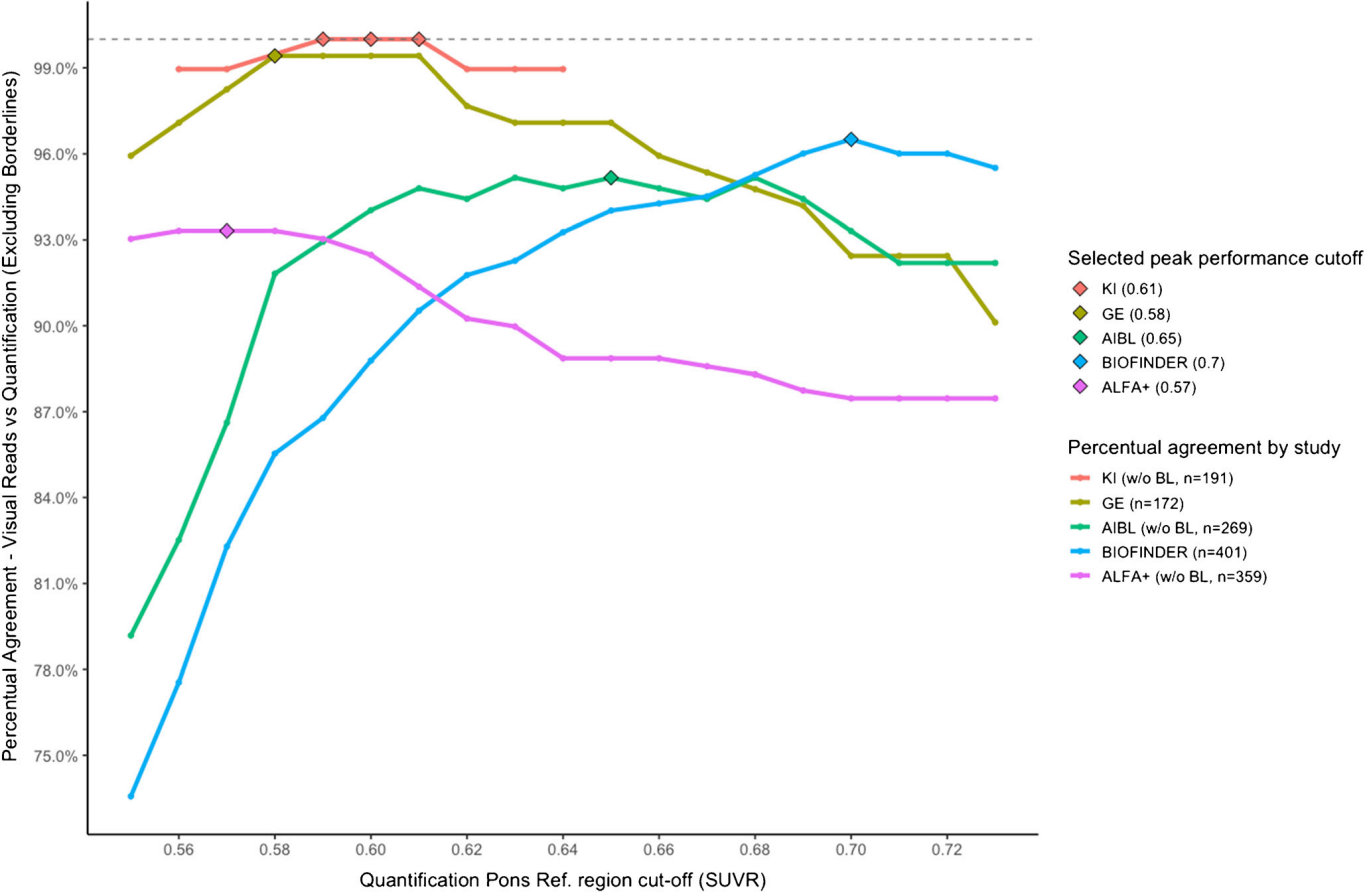
Change in % agreement between visual and quantitative image interpretation around the SUVr pons threshold of 0.55 to 0.74 (with borderlines (BL) excluded). Note: The number of BL cases excluded is 21, 2 and 7 for KAROLINSKA, ALFA+ and AIBL, respectively

Results shown in Fig. 1 indicate that, for all five studies considered, the optimal cut-off was reached when pons is used as the RR. Both the GE Healthcare and KAROLINSKA cohort had high agreement across the threshold range 0.56–0.64 (GE [97.1–99.4%], KAROLINSKA [98.4–100%]), probably reflected by the use of similar software packages using a comparable region of interest for the composite cortical area [17]. The AIBL group showed a relative high agreement across a wider range of cut-offs 0.59–0.7 [92.2–95.2%]. The BIOFINDER population (MCI) showed most of the agreement at highest cut-off threshold among the observed (0.7 [96%]) and then decreased steadily as the threshold decreased down to 0.55 with the lowest agreement assessed among the five cohorts [73.6%].

The ALFA+ cohort (consisting of a CU population) presented a curve skewed towards the lowest cut-offs ranges and had its selected optimal agreement at the lowest cut-off among the five studies (0.57 [93.3%]).

Compared to GE and KAROLINSKA, the other three studies (AIBL, ALFA+, BIOFINDER), used research-based software tools with varying trends in the sensitivity analysis observed. This may be due to different strategies used for the creation of the cortical region of interest which may have captured some spill-over of white matter from the non-specific PET signal influencing overall SUVr values as well. Another factor potentially contributing to differences among sensitivity curves is the amount and subtype of subjects in the range selected for sensitivity analysis testing. GE and KAROLINSKA had less than 25 subjects in the full range analysed, ALFA+, AIBL and BIOFINDER had respectively 32, 74 and 112 subjects in the full range (0.55–0.73) and the subtype of population with ALFA+ characterized by only HC (AD Offspring), AIBL including mostly HC (*n* = 59) and only few MCI (*n* = 10) and AD (*n* = 3), BIOFINDER included approximately equal amount of HC (*n* = 35), SCD (*n* = 38) and MCI (*n* = 39) in the range of exam.

### 4) Analysis of the discordant visual read and quantitative results of the pons reference region

**Fig. 2:**
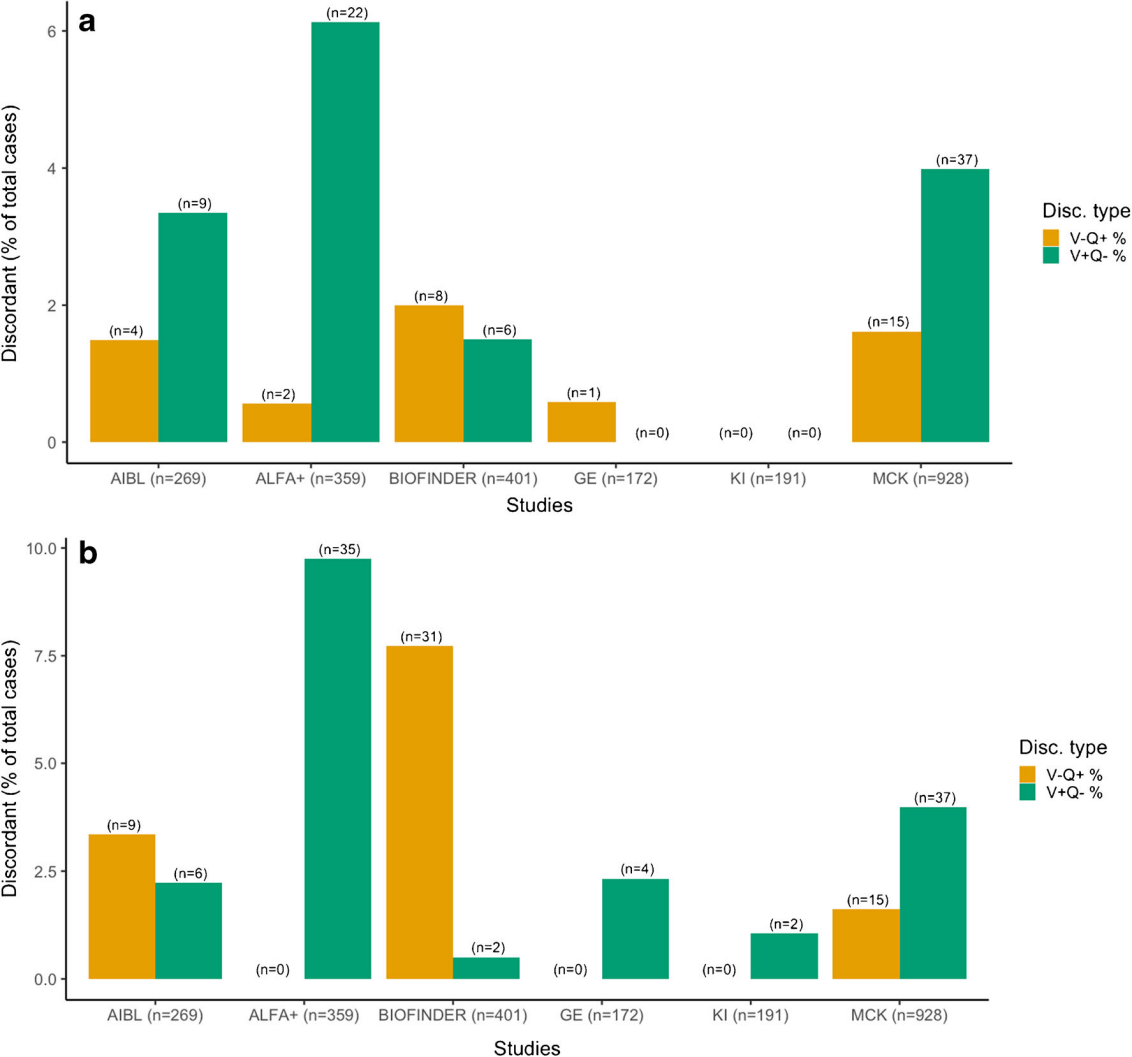
Representation of number of discordant cases by study with **a**) variable (maximal performance) pons cut-off and **b**) fixed cut-off (0.62) (both with borderlines excluded). Orange bars are visual negative/quantitation positive, green bars are visual positive/quantitation negative

Figure 2a, b highlights the relative distribution of discordant cases (V-Q+ or V+Q-) across the studies with pons as RR, using an optimized cut-off and fixed cut-off (0.62) (GE, KAROLINSKA, MCK, ALFA+, Biofinder, AIBL), respectively. Fisher’s exact test (*p* < 0.001) demonstrated differences between studies by discordant type using optimized cut-off with especially ALFA+ having a higher number of V+Q- (around 6% of the total cohort and 91.7% of all discordant cases in the cohort); using a fixed cut-off of 0.62, AIBL and BIOFINDER studies resulted having a higher number of V-Q+ (around 9% of the total cohort), whereas the other cohorts had a higher proportion of V+Q- discordant cases.

Across all studies, on average 4–5% of the images showed discordance between the visual inspection read and the quantitative measure (Table 3a, b). It must be noted that the absolute number of discordant cases is lower with variable cut-off compared to fixed cut-off. In the studies including more overt clinical cases (MCI, AD etc.), there was greater agreement in the visual vs quantification measures. The greatest relative proportion of discordant cases are seen in the research populations of ALFA+ (*n* = 35, 9.7%) and BIOFINDER (*n* = 33, 8.2%) suggesting that these subjects with emerging amyloid burden need more careful inspection and analysis. The two groups of discordant cases (V+Q- vs V-Q+) did not differ by gender, age, nor APOE-ε4 status when using the optimized cut-off for pons reference region. When using the fixed cut-off (0.62), V+Q- vs V-Q+ did not differ by gender but differ by age (67 ± 7 vs 73 ± 5, respectively, *p* < 0.001, range 55–91 y), and by APOE-ε4 status, in the sense that V+Q- had 67.4% (*n* = 29) of carriers compared to 11.1% (*n* = 1) for V-Q+, for the 51 discordant with this information (*p* = 0.002).

**Table 3:** a Summary of discordant and concordant scans when examining visual vs SUVr (Pons, optimized cut-off). b Summary of discordant scans when examining visual vs SUVr (Pons, 0.62 cut-off)

Study	Total cases	Total concordant V+Q+	Total concordant V-Q-	Total discordant	% Disc	Total borderline/Q+	Total borderline/Q-	Agreement
a								
GE	172	71	100	1	1%	-	-	99.4%
KAROLINSKA	207	94	97	0	0%	7	9	100%
MCK	928	634	242	52	6%	-	-	94.4%
ALFA+	361	24	311	24	7%	1	1	93.3%
BIOFINDER	401	117	270	14	3%	-	-	96.5%
AIBL	276	96	160	13	5%	2	5	95.2%
Total	2345	1036	1180	104	4% (mean)	10	15	94.4% (mean)
b								
GE	172	67	101	4	2%	-	-	97.7%
KAROLINSKA	207	92	97	2	1%	5	11	99.0%
MCK	928	634	242	52	6%	-	-	94.4%
ALFA+	361	11	313	35	10%	1	1	89.4%
BIOFINDER	401	121	247	33	8%	-	-	91.8%
AIBL	276	99	155	15	5%	4	3	94.4%
Total	2345	1024	1155	141	5% (mean)	10	15	94.4% (mean)

The agreement is calculated on the total number of cases excluding borderlines

Analysis of discordant V+Q- vs V-Q+ by diagnosis (Tables 4 and 5) with Fisher’s exact test showed differences between the two discordant groups and their diagnosis (*p* < 0.05), with SCD and HC groups over-represented in the V-Q+ discordant group using fixed cut-off (Table 5). The post hoc tests corrected for Bonferroni multiple comparison correction produced significant differences using fixed cut-off, in the sense that HC (AD offspring) have significantly higher number than V+Q- than SCD and MCI.

**Table 4:** Discordant visual read/quantitation cases with diagnostic information combined (Pons, optimized cut-off)

	V+Q- (*n* = 37)	V-Q+ (*n* = 14)	Total (*n* = 51)	*p* value
Diagnosis				0.014
HC	7 (50%)	7 (50%)	14	
HC(ADO)	22 (91.7%)	2 (8.3%)	24	
SCD	2 (40%)	3 (60%)	5	
MCI	4 (66.7%)	2 (33.3%)	6	
AD	1 (100%)	0 (0%)	1	
Non-AD	1 (100%)	0 (0%)	1	

*Abbreviations*: *HC*, healthy control; *ADO*, offspring of AD parent(s); *SCD*, subjective cognitive decline; *MCI*, mild cognitive impairement; *AD*, Alzheimer’s disease; *non-AD*, non-AD dementia

*p* value is from Fisher’s exact test. Post hoc with Bonferroni correction did not produce significant results

**Table 5:** Discordant visual read/quantitation cases with diagnostic information combined (Pons, 0.62 cut-off)

	V+Q- (*n* = 45)	V-Q+ (*n* = 40)	Total (*n* = 85)	*p* value
Diagnosis				<0.001
HC	2 (13.3%)	13 (86.7%)	15	
HC(ADO)	35 (100%)	0 (0%)	35	
SCD	2 (11.8%)	15 (88.2%)	17	
MCI	2 (15.4%)	11 (84.6%)	13	
AD	3 (75%)	1 (25%)	4	
Non-AD	1 (100%)	0 (0%)	1	

*Abbreviations*: HC, healthy control; ADO, Offspring of AD parent(s); SCD, subjective cognitive decline; MCI, mild cognitive impairement; AD, Alzheimer’s disease; non-AD, non-AD dementia

*p* value is from Fisher’s exact test. Post hoc with Bonferroni correction produced significant results for the comparisons HC(ADO) vs SCD and MCI (separately)

### 5) Clinical follow-up of discordant cases

#### Pons results (KAROLINSKA, BIOFINDER, AIBL)

Tables 6 and 7 show the discordant types (V+Q- and V-Q+) and Tables 8 and 9 show the borderline types (BL/Q- and BL/Q+) in relation to clinical progression from baseline for the three studies with follow-up data available until 4 years using the two different types of cut-off. No differences between discordant types were found when evaluating the progression to any diagnosis or the progression to AD/Other Diagnosis (OD) or the progression to specific diagnosis with chi-square analysis, regardless of the cut-off used.

**Table 6:** Follow-up data (up to 4 y) available for pons reference region (optimized cut-off) (only discordant cases)

	V-Q+ (*N* = 6)	V+Q- (*N* = 3)	Total (*N* = 9)	*p* value
Progression to any clinical diagnosis				0.134
Clinical progression	3 (50.0%)	3 (100%.0%)	6 (66.7%)	
Stable	3 (50.0%)	0 (0.0%)	3 (33.3%)	
Progression to AD/other diagnosis				0.223
Progression to AD	2 (33.3%)	1 (33.3%)	3 (33.3%)	
Progression to other diagnosis	1 (16.7%)	2 (66.7%)	3 (33.3%)	
Stable	3 (50.0%)	0 (0.0%)	3 (33.3%)	
Progression in detail				0.240
HD to SCD	0.(0.0%)	1 (33.3%)	1 (11.1%)	
MCI to AD	1 (16.7%)	1 (33.3%)	2 (22.2%)	
SCD to AD	1 (16.7%)	0 (0.0%)	1 (11.1%)	
SCD to MCI	0 (0.0%)	1 (33.3%)	1 (11.1%)	
SCD to Parkinsonian	1 (16.7%)	0 (0.0%)	1 (11.1%)	
Stable	3 (50.0%)	0 (0.0%)	3 (33.3%)

**Table 7:** Follow-up data (up to 4 y) available for pons reference region (0.62 fixed cut-off) (only discordant cases)

	V-Q+ (*N* = 21)	V+Q- (*N* = 4)	Total (*N* = 25)	*p* value
Progression to any clinical diagnosis				0.524
Clinical progression	14 (66.7%)	2 (50.0%)	16 (64.0%)	
Stable	7 (33.3%)	2 (50.0%)	9 (36.0%)	
Progression to AD/other diagnosis				0.322
Progression to AD	8. (38.1%)	0 (0.0%)	8 (32.0%)	
Progression to other diagnosis	6 (28.6%)	2 (50.0%)	8 (32.0%)	
Stable	7 (33.3%)	2 (50.0%)	9 (36.0%)	
Progression in detail				0.206
HD to SCD	1 (4.8%)	1 (25.0%)	2 (8.0%)	
MCI to AD	4 (19.0%)	0 (0.0%)	4 (16.0%)	
MCI to Parkinsonian	2 (9.5%)	0 (0.0%)	2 (8.0%)	
SCD to AD	4 (19.0%)	0 (0.0%)	4 (16.0%)	
SCD to MCI	0 (0.0%)	1 (25.0%)	1 (4.0%)	
SCD to Parkinsonian	1 (4.8%)	0 (0.0%)	1 (4.0%)	
SCD to vascular	2 (9.5%)	0 (0.0%)	2 (8.0%)	
Stable	7 (33.3%)	2 (50.0%)	9 (36.0%)

**Table 8:** Follow-up data (up to 4 y) available for pons reference region (optimized cut-off) (only BL cases)

	BL/Q- (*N* = 5)	BL/Q+ (*N* = 4)	Total (*N* = 9)	*p* value
Progression to any clinical diagnosis				0.343
Clinical progression	5 (80.0%)	4 (100.0%)	8 (88.9%)	
Stable	1 (20.0%)	0 (0.0%)	1 (11.1%)	
Progression to AD/other diagnosis				0.358
Progression to AD	4 (80.0%)	3 (75.0%)	7 (77.8%)	
Progression to other diagnosis	0 (0.0%)	1 (25.0%)	1 (11.1%)	
Stable	1 (20.0%)	0 (0.0%)	1 (11.1%)	
Progression in detail				0.358
HC to SCD	0 (0.0%)	1 (25.0%)	1 (11.1%)	
MCI to AD	4 (80.0%)	3 (75.0%)	7 (77.8%)	
Stable	1 (20.0%)	0 (0.0%)	1 (11.1%)

**Table 9:** Follow-up data (up to 4 y) available for pons reference region (0.62 fixed cut-off) (only BL cases)

	BL/Q- (*N* = 6)	BL/Q+(*N* = 3)	Total (*N* = 9)	*p* value
Progression to any clinical diagnosis				0.453
Clinical progression	5 (83.3%)	3 (100.0%)	8 (88.9%)	
Stable	1 (16.7%)	0 (0.0%)	1 (11.1%)	
Progression to AD/other diagnosis				0.276
Progression to AD	5 (83.3%)	2 (66.7%)	7 (77.8%)	
Progression to other diagnosis	0 (0.0%)	1 (33.3%)	1 (11.1%)	
Stable	1 (16.7%)	0 (0.0%)	1 (11.1%)	
Progression in detail				0.276
HC to SCD	0 (0.0%)	1 (33.3%)	1 (11.1%)	
MCI to AD	5 (83.3%)	2 (66.7%)	7 (77.8%)	
Stable	1 (16.7%)	0 (0.0%)	1 (11.1%)	

Nonetheless, according to competing risk regression analysis that took advantage of the full follow-up data (up to 7 years), using censoring similar to a survival analysis and discounting the contribution of the competing events (AD and OD progression), the V-Q+ discordant cases were 11% (CI 95%: 4–34%) more likely to progress to AD than V+Q- discordant cases (*p* < 0.001).

#### WC (SLC, AUMC, AIBL)

Analysis of WC results showed no differences in the two subtypes of discordancy or borderline when analysing for overall progression to OD as shown in Tables 10 and 11 but it must be noted that the sample available was limited and progression to AD presented too few cases for further analyses.

**Table 10:** Follow-up data (up to 3 yrs) available for WC reference region (only discordant cases)

	V-Q+ (*N* = 1)	V+Q- (*N* = 6)	Total (*N* = 7)	*p* value
Progression to any clinical diagnosis				0.350
Clinical progression	0 (0.0%)	3 (50.0%)	3 (42.9%)	
Stable	1 (100.0%)	3 (50.0%)	4 (57.1%)	
Progression to AD/otherwise diagnosis				0.646
Progression to AD	0 (0.0%)	1 (16.7%)	1 (14.3%)	
Progression to other diagnosis	0 (0.0%)	2 (33.3%)	2 (28.6%)	
Stable	1 (100.0%)	3 (50.0%)	4 (57.1%)	
Progression in detail				0.831
HC to SCD	0 (0.0%)	1 (16.7%)	1 (14.3%)	
MCI to AD	0 (0.0%)	1 (16.7%)	1 (14.3%)	
SCD to MCI	0 (0.0%)	1 (16.7%)	1 (14.3%)	
Stable	1 (100.0%)	3 (50.0%)	4 (57.1%)

**Table 11:** Follow-up data (up to 3 yrs) available for WC reference region (only BL cases)

	BL/Q- (*N* = 3)	BL/Q+ (*N* = 3)	Total (*N* = 6)	*p* value
Progression to any clinical diagnosis				0.273
Clinical progression	0 (0.0%)	1 (33.3%)	1 (16.7%)	
Stable	3 (100.0%)	2 (66.7%)	5 (83.3%)	
Progress to AD/other diagnosis				0.273
Progression to other diagnosis	0 (0.0%)	1 (33.3%)	1 (16.7%)	
Stable	3 (100.0%)	2 (66.7%)	5 (83.3%)	
Progression in detail				0.273
HC to SCD	0 (0.0%)	1 (33.3%)	1 (16.7%)	
Stable	3 (100.0%)	2 (66.7%)	5 (83.3%)	

### 6) PET image reconstruction parameter analysis

A high correlation between the reference reconstruction (Ref 128 × 128 matrix) and other reconstruction methods (Alt-256: *R*^2^ = 0.99, *p* < 0.0001 and Alt-400: *R*^2^ = 0.95, *p* < 0.0001) was observed (Supp Fig. 1 a, b). Bland-Altman plots indicated there were insignificant changes in individual SUVr results between reconstruction methods over the SUVr range examined (Supp Fig. 2 a, b) indicating the processing of [^18^F]flutemetamol images acquired at approx. 90 min post injection for 20 min of acquisition is reasonably robust. Supp Fig. 3 shows the sensitivity analysis plot for the ALFA+ study performed with two different pipelines (based on AAL and CTX atlases). The agreement has different trends due to the different target regions, with CTX being more affected by white matter and having on average higher SUVr values that shift the optimal cut-off to the right of the probed range of cut-offs. Despite these differences, it can be observed that using the pre-specified threshold of 0.62 the agreement against visual reads with either of the two pipelines is very high (>90%), thus supporting the validity of the approach in this work, irrespective of absolute differences in SUVr values that may arise from different quantification pipelines or image reconstruction settings.

## Discussion

In this multicentre study including over 2700 scans, the binary visual inspection of [^18^F]flutemetamol had a very high agreement to a composite quantified measure of brain amyloid with the majority of analysis reporting a 93–99% agreement. This was observed irrespective of the reference region used or processed by a multitude of software tools (some CE marked/510K approved and others used for research purposes only) showing the high generalizability/utility of the two measures when considered for a pathology threshold relative to current clinical practice [6, 17].

Knowing that the accuracy of visual inspection is high, the value of quantitation lies in its ability to support image interpretation in cases where there is a lower confidence in the read, for example where the amyloid deposition may be close to the pathology threshold or for readers who may lack experience in routine image interpretation or for those readers who process scans on a less frequent basis. Quantitation could also provide future value beyond supporting simple dichotomy of image interpretation [37] if developing levels of amyloid become clinically relevant for therapy intervention or monitoring [38].

Quantitative software can provide information about the regional distribution of amyloid, the comparison of an individual case to a range of normal cases (using *Z* scores) and regional and composite standard uptake volume ratios (SUVrs) or Ab_Load_ (in the case of Amyloid^IQ^). Both the latter techniques would be most similar to a binary visual read. Much data has been accumulated since the approval of [^18^F]flutemetamol and other amyloid PET tracers, to show the concordance between visual inspection and quantitative methods using both CE marked software and other widely available processing tools.

The use of continuous quantitative cortical amyloid measures (both as composite and regional assessments) is long practiced in the research setting but is less well characterized as an adjunct to the approved visual reading methods practiced for clinical routine. More recently studies such as that from Chincarini et al. [14] have begun to assess how quantitative assessment can supplement the dichotomous visual read of amyloid PET in particular to give confidence to read situations where the scan could be classified as mildly negative or positive or to borderline cases.

There was discordancy in the results in approximately 4–5% of cases (with pons reference region), with higher agreement rates found in general in those subjects which had been included in routine clinical practice rather than those in research, whose amyloid would have been evolving rather than established.

There was some variability observed when analysing the cohorts in more detail and performing the sensitivity analysis to find optimal cut-off values. One explanation for the variability of the cut-offs seen between the cohorts may be due to differing strategies around the composition of the cortical regions of interest used for the SUVr analysis. If some spill-over of white matter signal is captured, this could lead to increased SUVr values for example as seen in BIOFINDER, whereas software using a more conservative ‘narrow’ mask could maximize the distance between white and grey matter border with resulting lower SUVr values. Additionally, the number and composition of subjects in the region of the sensitivity range would influence the analysis.

The follow-up information evidenced that in our dataset of discordant subjects in the pons region, there were similar proportions which either remained stable over time or progressed to any clinical diagnosis. Interestingly, in the dataset with no borderlines, the discordant type V-Q+ was 11% more likely to progress to AD than V+Q-, indicating, even with a relatively small effect, that in case of discordance between visual read and quantitation, the quantitation provides the positivity relevant to progression for AD. This was true even without taking into account of regionality that most likely would increase the value of the discrimination of the grey zone.

Practical experience from the KAROLINSKA site indicates that in general the majority of [^18^F]flutemetamol scans have a straightforward uptake pattern based on visual reading alone. A minor number of scans, however, were found to be borderline primarily when the cortical tracer uptake is not global but appears in only few regions, such as the precuneus/posterior cingulate or frontal cortex, or sometimes the pattern is uncertain due to cortical atrophy. Due to the high impact an amyloid PET may have on the final diagnosis and treatment, a definite conclusion of positivity/negativity of amyloid scan is however highly desirable.

**Fig. 3:**
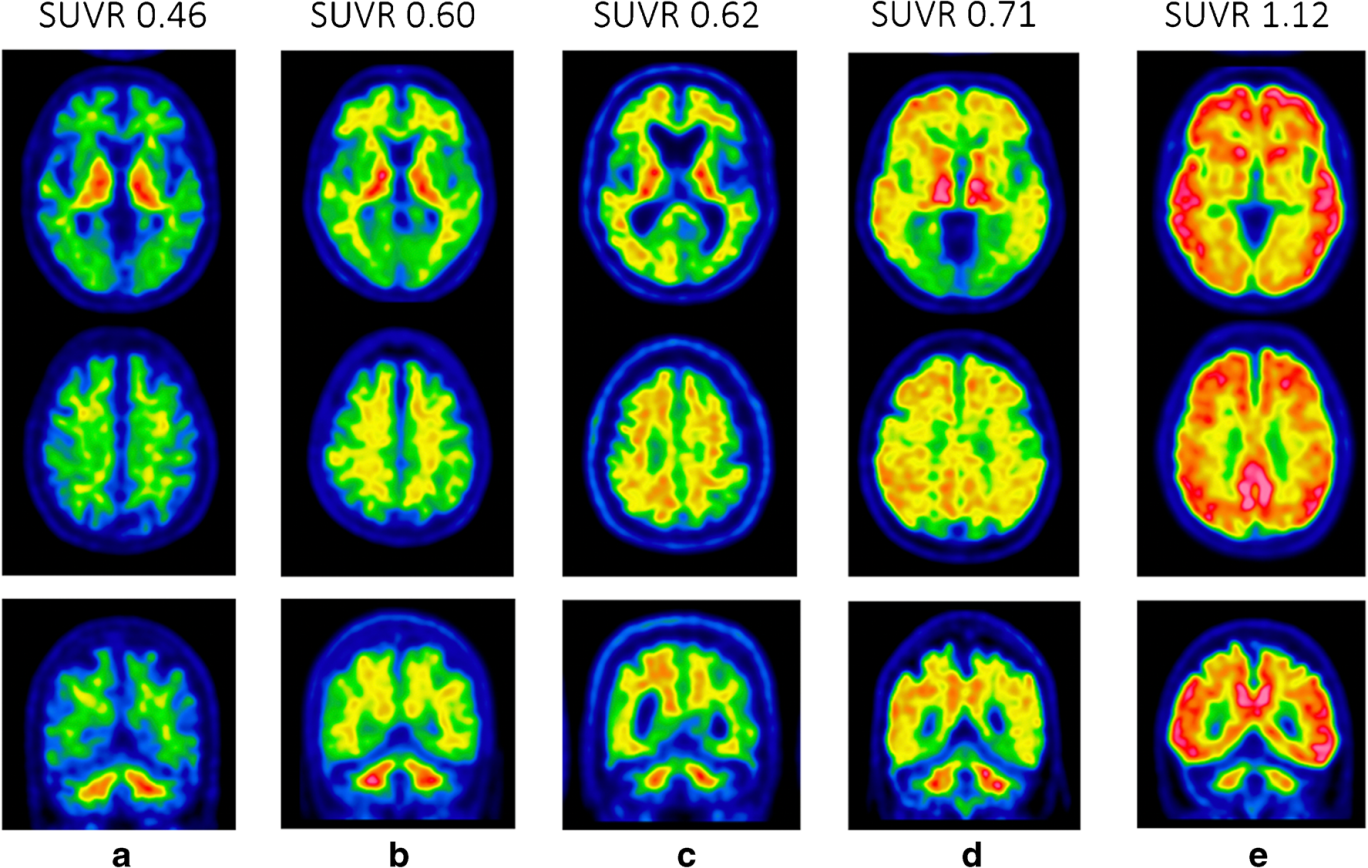
Transaxial images at the level of striatum and the upper level of the brain as well as coronal image at the posterior cingulate/precuneus level (top, middle and bottom row, resp). **a** shows a clear negative scan, whilst **d** and **e** show clear positive scans with different level of diffuse flutemetamol uptake in the brain cortex and striatum. **b** and **c** present borderline cases with possible uptake in the precuneus/posterior cingulate (**b**) and asymmetrically enhanced cortical uptake on the right side (**c**), both reported as possible positive scans (note patient in **b** showed a clearer positive finding at follow-up scan some years later)

In this sense, quantification of amyloid uptake as a second opinion can supplement the visual appreciation and enhance confidence in the image result. This support is also valuable in cases with definite uptake patterns to increase the output of reads in the nuclear medicine department but is even more important for visual read cases close to the pathology threshold. Examples of borderline cases (see Fig. 3) show quantitative values close to the threshold and in these cases; quantification was able either to make a conclusion or to report the method limitations and report true uncertainty.

### Generic discussion on the use of quantitative methods in amyloid imaging

From a practical perspective, there are some general points to remember when performing quantitation to supplement visual reading. Volume of interest placement over cortical regions needs to be accurate [39] and the influence of atrophy could dilute the value of the PET measure [40]. Enlarged ventricles cause thinning of the cortical ribbon (and hence the CT or MRI scan should also be assessed) whilst local uptake in a single region (for example elevated PET uptake in striatum has been seen in presenilin cases [41]) could be missed in an overall composite cortical measure. Finally, amyloid levels close to the threshold may render the scan difficult to interpret using visual inspection alone [5]. In this instance quantitation may be used to support the read although it is suggested that systematic careful visual inspection should be performed with the above points in mind.

Whilst simple dichotomous reads are currently the standard and sufficient in clinical routine, recent work has shown the possible value of assessing amyloid burden beyond the binary classification of scans and the possibility to use different SUVr cut-off values for different clinical questions [22, 42]. In addition to SUVr measures (as well as *Z* scores when a normal database is included), the Centiloid (CL) measure is now standard in research practice [43] and studies have identified specific cut-offs for emerging amyloid pathology (CL~12) compared to *post-mortem* [44] established AD pathology (~30) compared to CSF measures [22]. The CL threshold for visual inspection of [^18^F]flutemetamol was shown to be approximately 40 units in a clinical study with MCI subjects [20]. In relation to predicting cognitive decline, the AIBL team followed a healthy volunteer cohort and found increasing CLs from 25 to over 100 corresponded to a ten-fold increased risk of progression to MCI over a 5-year observation period [25]. The St Luc team had an equally long follow-up period of 6 years with a CL level of 26 optimally predicting progression to dementia from earlier clinical stages [20]. Considering these different CL cut-offs and their possible clinical value, it is of interest to investigate the value of regional visual assessment (i.e. number of positive regions) and their respective CL burden, to further optimize the utility of [^18^F]flutemetamol reads in the clinical routine. Taken together, it would be valuable to collate more cohorts with available regional visual assessment and long-term clinical follow-up in order to more accurately predict the value of baseline amyloid status to future cognitive decline with the aim of more optimal patient management. More novel techniques for image interpretation such as machine learning algorithms could also be implemented [45, 46] although a most recent study [46] still reports a discordant rate of 8% between the visual read and the automated read with a large sample size of over 330 subjects.

### Limitations

This study is not without limitations. The SUVr unit (or Ab_Load_ for Amyloid^IQ^) was used rather than the more recently introduced Centiloid measure which is now routine for research studies [43]. Since the majority of CE marked amyloid PET image analysis tools do not yet analyse amyloid levels in Centiloids, we used SUVr or Ab_Load_ for our analysis, particularly as the analysis was focused upon a single PET tracer [^18^F]flutemetamol and the information contained in clinical work. It would have been preferable too to include more image concordance data too from routine use, though these cohorts are not frequently available because the advent of quantification has only recently been introduced into routine practice. Hence, we used data from a selection of other studies, some of which used MCI and AD cases which would have advanced and widespread amyloid deposition, but also from some cohorts which included at risk of AD and subjective cognitive decline which might have ‘developing’ amyloid and hence introduce some variability into the analysis. Although we showed that changing the PET reconstruction settings for a single camera did not impact the SUVr measure, we cannot assume this would be consistent across the multitude of camera types used and hence multisite initiatives such as AMYPAD (www.amypad.eu) are systematically examining this in further detail. The impact of camera types, reconstruction settings and the fit of the cortical mask for assessing the composite amyloid load may be even more critical if small longitudinal changes in amyloid load are measured for therapy monitoring [40] or if subthreshold levels of amyloid are required for early target engagement approaches with new therapies [47].

Some of the studies reported ‘borderline’ cases where an exact negative or positive read result was not recorded. The fraction of this borderlines compared to the total number of cases was <1% and they were excluded from cross-sectional analyses as inclusion as either negative or positive did not impact the overall results.

The comparison of the different reconstruction parameters highlighted the relative stability of the SUVr measurement for the pons region and justified pooling together different studies with slightly different methods of reconstruction. Regardless, the pooled statistics were always assessed within each study first and then the aggregated measures pooled together (i.e. number of discordant cases). Finally, it is not possible to comment on the comparative performance of the different quantitative approaches because of the high agreement of all methods with visual reads and a number of important factors that varied by study, e.g. different subject populations, different readers and processing pipelines with and without adjunct MR data. Further studies involving application of the different analytical pipelines to the same dataset would be required in order to elucidate any more subtle differences in performance between the approaches.

## Conclusion

In summary, quantitation of composite brain amyloid shows a very high agreement (~93–99%) vs binary visual reading and also allows for a continuous measure that could be used in the future to qualify subthreshold levels or monitor disease progression and be indicative or predictive of future cognitive decline. From a routine clinical perspective, the physician could choose to use CE marked software tools to supplement their read methodology but should still retain the careful inspection of an image by visual means. Analysis of discordancy and the added value of considering visually negative and quantitative positive cases as potentially more at risk adds a tool to the routine evaluations. The future utility of PET amyloid quantitation could also be valuable for identifying early amyloid deposition if therapy intervention in preclinical cases becomes a reality. There also might be value in standardized approaches for image quantitation methodology particularly for cases which are close to the amyloid pathology threshold.

## Supplementary information

ESM 1 (PDF 561 kb)

## Declarations

Data described in this report was compiled post hoc (by MB) from the clinical cohorts described in references [17–23, 26–28]. Each of the studies reported was conducted with the principles of the Declaration of Helsinki, the Good Clinical Practise: Consolidated Guideline approved by the International Conference on Harmonisation and applicable national and local laws and regulations. The Ethical boards for the specific studies are listed: GE: statement of ethics compliance (and committees) in Wolk et al. 2018 [48]; KAROLINSKA: Ethical approval was obtained from the Regional Human Ethical Committee of Stockholm (2016-120-32); MCK: statement of ethics compliance in Sur et al. 2020 [19]; Le Comité d’Ethique Hospitalo-Facultaire (CEHF) des Cliniques Universitaires Saint-Luc (CUSL) approved the project from SLC under the number UCL-2010-412 (Eudra-CT: 2011-001756-12); VU University Medical Center approved the Dutch Flutemetamol study (AUMC) (reference number: 2012/302); Parc de Salut Mar (BBRC, Barcelona); BIOFINDER/INVICRO (Lund Ethics Committee, Sweden); Austin Health Human Research Ethics Committee approved the AIBL study.

## References

[1] European Medicines Agency. Vizamyl: summary of product characteristics. London: European Medicines Agency; 2017. https://www.ema.europa.eu/en/documents/product-information/vizamyl-epar-product-information_en.pdf. Accessed 14 Aug 2020.

[2] GE Healthcare. Vizamyl: prescribing information. Arlington Heights, IL: GE Healthcare; 2017. https://www.accessdata.fda.gov/drugsatfda_docs/label/2017/203137s008lbl.pdf. Accessed 14 Aug 2020.

[3] Chételat G, Arbizu J, Barthel H, Garibotto V, Law I, Morbelli S (2020). Amyloid-PET and 18F-FDG-PET in the diagnostic investigation of Alzheimer’s disease and other dementias. The Lancet Neurology.

[4] Mirra SS, Heyman A, McKeel D, Sumi SM, Crain BJ, Brownlee LM (1991). The Consortium to Establish a Registry for Alzheimer’s Disease (CERAD). Part II. Standardization of the neuropathologic assessment of Alzheimer’s disease. Neurology..

[5] Ikonomovic MD, Buckley CJ, Heurling K, Sherwin P, Jones PA, Zanette M (2016). Post-mortem histopathology underlying β-amyloid PET imaging following flutemetamol F 18 injection. Acta Neuropathol Commun.

[6] Salloway S, Gamez JE, Singh U, Sadowsky CH, Villena T, Sabbagh MN (2017). Performance of [18F] flutemetamol amyloid imaging against the neuritic plaque component of CERAD and the current (2012) NIA-AA recommendations for the neuropathologic diagnosis of Alzheimer’s disease. Alzheimer’s & Dementia: Diagnosis, Assessment & Disease Monitoring.

[7] Vandenberghe R, Van Laere K, Ivanoiu A, Salmon E, Bastin C, Triau E (2010). 18F-flutemetamol amyloid imaging in Alzheimer disease and mild cognitive impairment: a phase 2 trial. Ann Neurol.

[8] Buckley CJ, Sherwin PF, Smith AP, Wolber J, Weick SM, Brooks DJ (2017). Validation of an electronic image reader training programme for interpretation of [18F]flutemetamol β-amyloid PET brain images. Nucl Med Commun.

[9] GE Healthcare. Vizamyl™ flutemetamol 18F injection electronic training programme. Arlington Heights, IL: GE Healthcare; 2020. https://www.readvizamyl.com/en-gb. Accessed 14 Aug 2020.

[10] Farrar G, Molinuevo JL, Zanette M (2019). Is there a difference in regional read [18 F] flutemetamol amyloid patterns between end-of-life subjects and those with amnestic mild cognitive impairment?. Eur J Nucl Med Mol Imaging.

[11] Fantoni ER, Chalkidou A, O’Brien JT, Farrar G, Hammers A (2018). A systematic review and aggregated analysis on the impact of amyloid PET brain imaging on the diagnosis, diagnostic confidence, and management of patients being evaluated for Alzheimer’s disease. J Alzheimers Dis.

[12] Barthel H, Sabri O (2017). Clinical use and utility of amyloid imaging. J Nucl Med.

[13] European Medicines Agency. Vizamyl. European Medicines Agency; 2020. https://www.ema.europa.eu/en/medicines/human/EPAR/vizamyl. Accessed 14 Aug 2020.

[14] Chincarini A, Peira E, Morbelli S, Pardini M, Bauckneht M, Arbizu J (2019). Semi-quantification and grading of amyloid PET: a project of the European Alzheimer’s Disease Consortium (EADC). Neuroimage Clin.

[15] Pontecorvo MJ, Arora AK, Devine M, Lu M, Galante N, Siderowf A (2017). Quantitation of PET signal as an adjunct to visual interpretation of florbetapir imaging. Eur J Nucl Med Mol Imaging.

[16] Fakhry-Darian D, Patel NH, Khan S, Barwick T, Svensson W, Khan S (2019). Optimisation and usefulness of quantitative analysis of 18F-florbetapir PET. Br J Radiol.

[17] Thurfjell L, Lilja J, Lundqvist R, Buckley C, Smith A, Vandenberghe R (2014). Automated quantification of 18F-flutemetamol PET activity for categorizing scans as negative or positive for brain amyloid: concordance with visual image reads. J Nucl Med.

[18] Leuzy A, Savitcheva I, Chiotis K, Lilja J, Andersen P, Bogdanovic N (2019). Clinical impact of [18 F] flutemetamol PET among memory clinic patients with an unclear diagnosis. Eur J Nucl Med Mol Imaging.

[19] Sur C, Kost J, Scott D, Adamczuk K, Fox NC, Cummings JL, et al. BACE inhibition causes rapid, regional, and non-progressive volume reduction in Alzheimer’s disease brain. Brain. 2020. 10.1093/brain/awaa332. 10.1093/brain/awaa332 PMC8453290 33253354

[20] Hanseeuw BJ, Malotaux V, Dricot L, Quenon L, Sznajer Y, Cerman J, et al. Defining a Centiloid scale threshold predicting long-term progression to dementia in patients attending the memory clinic: an [Eur J Nucl Med Mol Imaging. 2020. doi:10.1007/s00259-020-04942-4. 10.1007/s00259-020-04942-4 PMC7835306 32601802

[21] Zwan MD, Bouwman FH, Konijnenberg E, Van Der Flier WM, Lammertsma AA, Verhey FR (2017). Diagnostic impact of [18 F] flutemetamol PET in early-onset dementia. Alzheimers Res Ther.

[22] Salvadó G, Molinuevo JL, Brugulat-Serrat A, Falcon C, Grau-Rivera O, Suárez-Calvet M (2019). Centiloid cut-off values for optimal agreement between PET and CSF core AD biomarkers. Alzheimers Res Ther.

[23] Hansson O, Seibyl J, Stomrud E, Zetterberg H, Trojanowski JQ, Bittner T (2018). CSF biomarkers of Alzheimer’s disease concord with amyloid-β PET and predict clinical progression: a study of fully automated immunoassays in BioFINDER and ADNI cohorts. Alzheimers Dement.

[24] Whittington A, Gunn RN (2019). Initiative AsDN. amyloid load: a more sensitive biomarker for amyloid imaging. J Nucl Med.

[25] van der Kall LM, Truong T, Burnham SC, Doré V, Mulligan RS, Bozinovski S, et al. Association of β-amyloid level, clinical progression and longitudinal cognitive change in normal older individuals. Neurology. 2020. 10.1212/WNL.0000000000011222. 10.1212/WNL.0000000000011222 PMC7884996 33184233

[26] Sur C, Klein G, Mukai Y, Mo Y, Voss T, Zhang Y, et al. Baseline amyloid PET imaging characteristics in Verubecestat (MK-8931) prodromal trial. *Conference Abstract* Human Amyloid Imaging. Florida, Miami; 2016.

[27] Molinuevo JL, Gramunt N, Gispert JD, Fauria K, Esteller M, Minguillon C (2016). The ALFA project: a research platform to identify early pathophysiological features of Alzheimer’s disease. Alzheimers Dement (N Y).

[28] Ellis KA, Bush AI, Darby D, De Fazio D, Foster J, Hudson P (2009). The Australian Imaging, Biomarkers and Lifestyle (AIBL) study of aging: methodology and baseline characteristics of 1112 individuals recruited for a longitudinal study of Alzheimer’s disease. Int Psychogeriatr.

[29] Bourgeat P, Villemagne VL, Dore V, Brown B, Macaulay SL, Martins R (2015). Comparison of MR-less PiB SUVR quantification methods. Neurobiol Aging.

[30] Hermes Medical Solutions. BRASS™. Stockholm, Sweden: Hermes Medical Solutions; 2000–2019 (C). https://www.hermesmedical.com/neurology/. Accessed 14 Aug 2020.

[31] Laboratory for Computational Neuroimaging Freesurfer. Athinoula A. Martinos Center for Biomedical Imaging; 2013. https://surfer.nmr.mgh.harvard.edu. Accessed 14 Aug 2020.

[32] LLC PT. PMOD. PMOD Technologies LLC; 2003. https://www.pmod.com/web/. Accessed 14 Aug 2020.

[33] Unit NR. PVElab. Copenhagen University Hospital; 2004. https://nru.dk/index.php/misc/category/37-pvelab. Accessed 14 Aug 2020.

[34] Wellcome Centre for Human Neuroimaging. Statistical Parametric Mapping (SPM) 12. University College London; 2020. http://www.fil.ion.ucl.ac.uk/spm/ Accessed 14 Aug 2020.

[35] Invicro. Amyloid Load (Amyloid^IQ^). 2018. https://invicro.com/case-studies/amyloid-load/. Accessed 14 Aug 2020.

[36] Donoghoe MW, Gebski V (2017). The importance of censoring in competing risks analysis of the subdistribution hazard. BMC Med Res Methodol.

[37] Peira E, Grazzini M, Bauckneht M, Sensi F, Bosco P, Arnaldi D, et al. Probing the role of a regional quantitative assessment of amyloid PET. J Alzheimers Dis. 2021. 10.3233/JAD-201156. 10.3233/JAD-201156 33554908

[38] Sperling R, Mormino E, Johnson K (2014). The evolution of preclinical Alzheimer’s disease: implications for prevention trials. Neuron..

[39] Chincarini A, Sensi F, Rei L, Bossert I, Morbelli S, Guerra UP (2016). Standardized uptake value ratio-independent evaluation of brain amyloidosis. J Alzheimers Dis.

[40] Schmidt ME, Chiao P, Klein G, Matthews D, Thurfjell L, Cole PE (2015). The influence of biological and technical factors on quantitative analysis of amyloid PET: points to consider and recommendations for controlling variability in longitudinal data. Alzheimers Dement.

[41] Klunk WE, Price JC, Mathis CA, Tsopelas ND, Lopresti BJ, Ziolko SK (2007). Amyloid deposition begins in the striatum of presenilin-1 mutation carriers from two unrelated pedigrees. J Neurosci.

[42] Habert MO, Bertin H, Labit M, Diallo M, Marie S, Martineau K (2018). Evaluation of amyloid status in a cohort of elderly individuals with memory complaints: validation of the method of quantification and determination of positivity thresholds. Ann Nucl Med.

[43] Klunk WE, Koeppe RA, Price JC, Benzinger TL, Devous MD, Jagust WJ (2015). The Centiloid Project: standardizing quantitative amyloid plaque estimation by PET. Alzheimers Dement.

[44] La Joie R, Ayakta N, Seeley WW, Borys E, Boxer AL, DeCarli C (2019). Multisite study of the relationships between antemortem [11C]PIB-PET Centiloid values and postmortem measures of Alzheimer’s disease neuropathology. Alzheimers Dement.

[45] Vandenberghe R, Nelissen N, Salmon E, Ivanoiu A, Hasselbalch S, Andersen A (2013). Binary classification of ^18^F-flutemetamol PET using machine learning: comparison with visual reads and structural MRI. Neuroimage..

[46] Kim JP, Kim J, Kim Y, Moon SH, Park YH, Yoo S (2020). Staging and quantification of florbetaben PET images using machine learning: impact of predicted regional cortical tracer uptake and amyloid stage on clinical outcomes. Eur J Nucl Med Mol Imaging.

[47] Leal SL, Lockhart SN, Maass A, Bell RK, Jagust WJ (2018). Subthreshold amyloid predicts tau deposition in aging. J Neurosci.

[48] Wolk DA, Sadowsky C, Safirstein B, Rinne JO, Duara R, Perry R (2018). Use of flutemetamol F 18-labeled positron emission tomography and other biomarkers to assess risk of clinical progression in patients with amnestic mild cognitive impairment. JAMA Neurol.

