# Supplementary material for: A multisite analysis of the concordance between visual image interpretation and quantitative analysis of [18F]flutemetamol amyloid PET images
Source: Eur J Nucl Med Mol Imaging. 2021 Apr 12;48(7):2183–99. doi: 10.1007/s00259-021-05311-5 (PMC8175298; doi:10.1007/s00259-021-05311-5)

Supplementary table 1 – Description of the reconstruction parameters by study

Study_abbr.	Scanner model	Recon type	Recon parms	Recon options	Matrix/Vox size	PVC	Frames
GE	1) SIEMENS Biograph CT<br>2) ECAT EXACT HR+<br>3) Discovery RX<br>4) Discovery STE<br>(multicenter)	1) 2D OSEM<br>2) FBP<br>3) 3D FBP<br>4) 3D OSEM	1a) 84 updates<br>1b) 96 updates	1) High Res<br>2,3) 3 mm post filter	128x128<br>1) 81 slices	No	20 min dyn (4 x 5 min)
KAROLINSK A	Siemens Biograph mCT PET/CT	3D OSEM	5 it, 21 subsets	PSF, TOF,<br>zoom factor 2<br>Gaussian filter:<br>1,2) 3 mm, 3) 2 mm	1) 128x128,<br>[2) 256x256,<br>3) 400x400<br>for comparative analysis]	No	20 min list-mode<br>90 min postinjection
MCK	PET and PET/CT scanners from GE, Siemens and Philips<br>(multicenter)	3D OSEM<br>(when possible)	(multicenter)	correction for attenuation (CT-based when possible)<br>(multicenter)	(multicenter)	No	(multicenter)
SLC	Philips Gemini PET/CT TF TOF 16 or 64<br>(64 slices after 2016)	LOR-RAMLA	2 it, 33 subsets	TOF	128x128/isometric 2 mm	No	30 min dyn (6 x 5 min)<br>90 min postinjection
AUMC	Philips Gemini TF PET/CT 64	LOR-RAMLA	2 it, 33 subsets	No PSF, no TOF	128x128x90	No	20 min dyn (4x 5 min)
ALFA+	Siemens Biograph mCT PET/CT	3D OSEM	8 it, 21 subsets	PSF, TOF	400x400/1.02x1.02x2.03mm	No	20 min dyn (4 x 5 min)
BIOFINDER	Philips Gemini	LOR-RAMLA	3 it, 33 subsets	-	128×128×90<br>slice thickness 2 mm	No	20 min dyn (4 x 5 min)<br>90 min postinjection
INVICRO	Philips Gemini	LOR-RAMLA	3 it, 33 subsets	-	128×128×90<br>slice thickness 2 mm	No	20 min dyn (4 x 5 min)<br>90 min postinjection
AIBL	1) Siemens PET/CT mCT 128<br>2) Philips Allegro PET (only 22 subjects)<br>3)Discovery 710 (72 subjects)	1) 3D OSEM<br>2) 3D-RAMLA<br>3) VPHD	1) 4it, 4 subsets<br>2) brain protocol<br>3) 3it, 16 subsets	1) no TOF<br>2) Sharp<br>3) 5mm post filter, Z axis filter light	1) 1.99 x 199x3.0<br>2) 2.0x2.0x2.0<br>3) 1.17x1.17x3.27	No	20 min dyn (4 x 5 min)<br>90 min postinjection

Supplementary Figure 1 – Correlation between Reference reconstruction parameters (Ref-128) and Alt-256 (a) and Alt-400 (b)

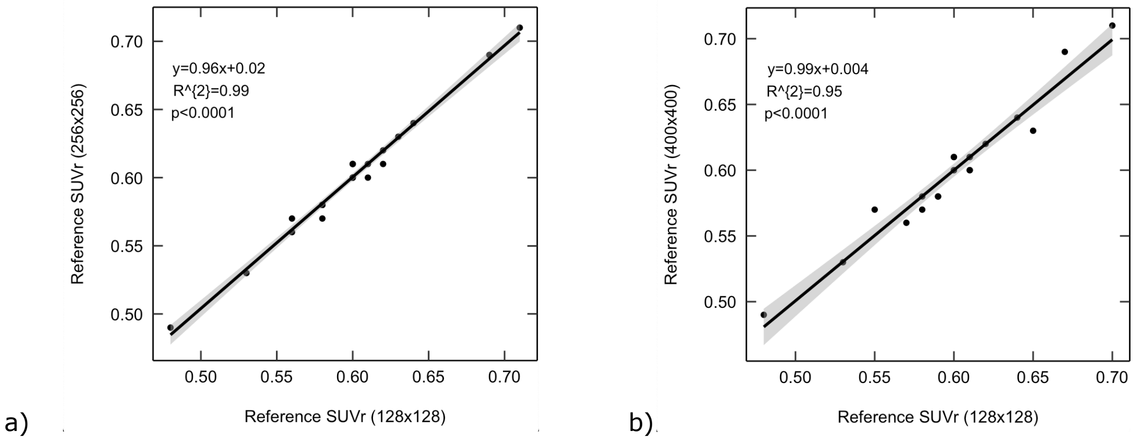

Supplementary Figure 2 – Bland-Altman plot between Reference reconstruction parameters (Ref-128) and Alt-256 (a) and Alt-400 (b)

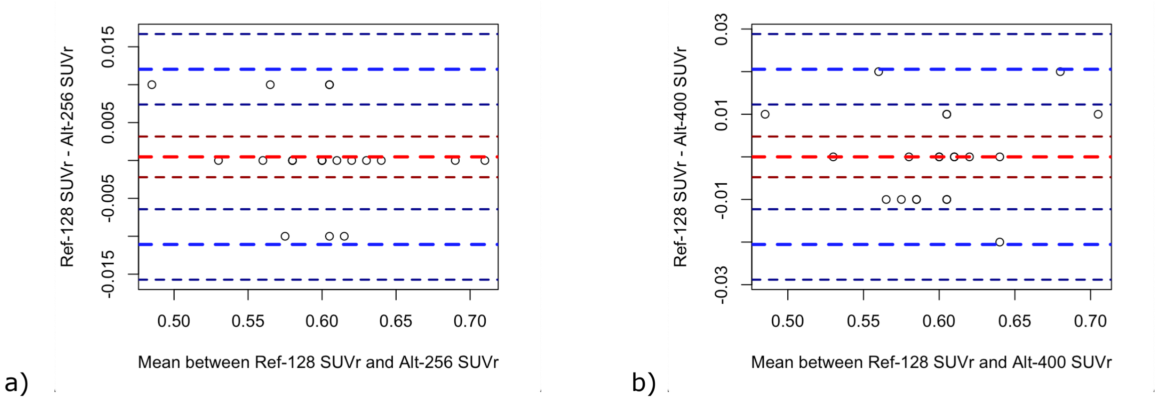

Supp Figure 3 Sensitivity analysis of Percentual Agreement with Pons reference region in the ALFA+ cohort with two different pipelines

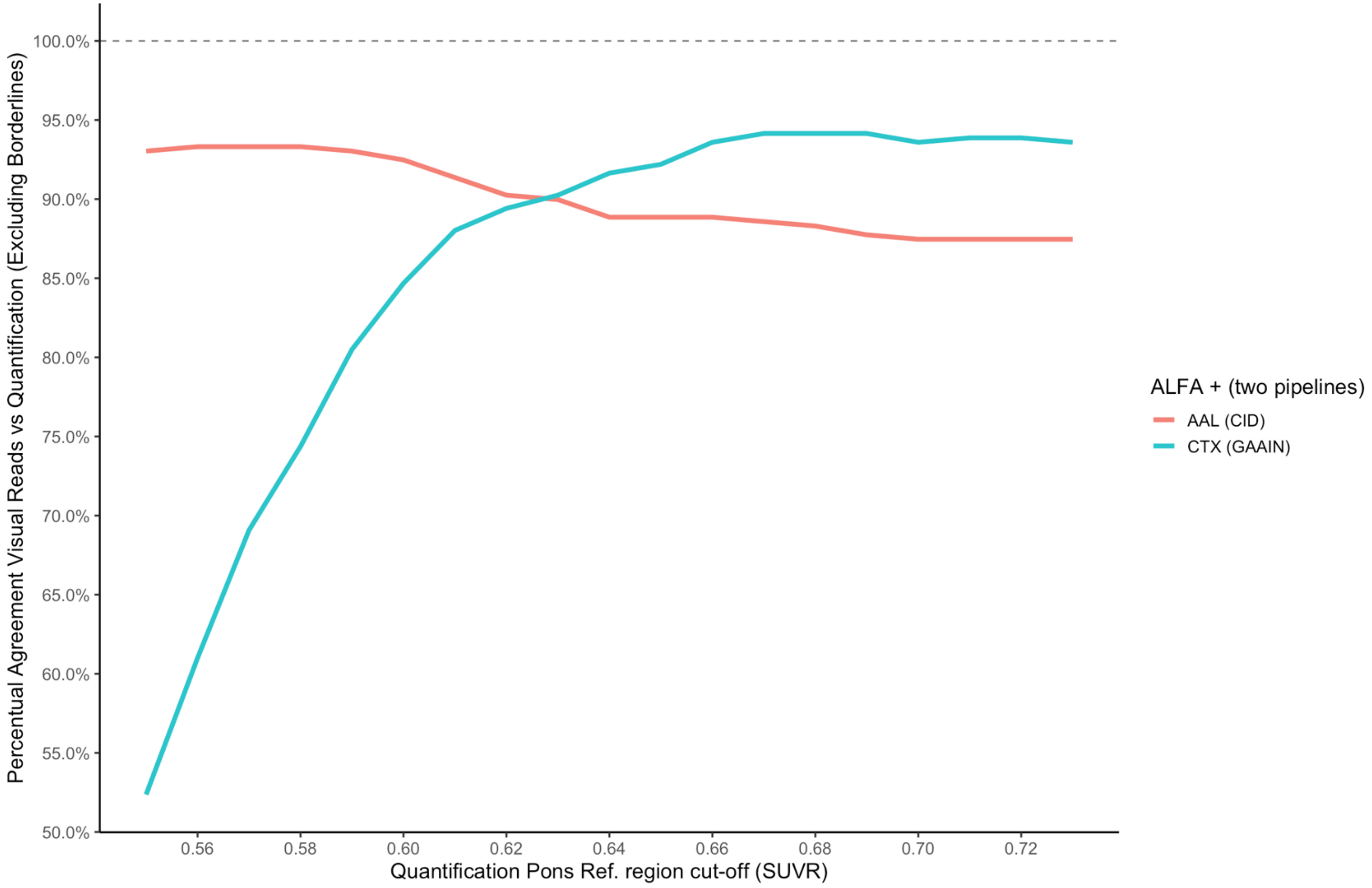

Supplement: Supplementary file 1 — (PDF 561 kb) [file 259_2021_5311_MOESM1_ESM.pdf]
